# Supplementary material for: Movement behavior remains stable in stroke survivors within the first two months after returning home
Source: PLoS One. 2020 Mar 26;15(3):e0229587. doi: 10.1371/journal.pone.0229587 (PMC7098564; doi:10.1371/journal.pone.0229587)
Supplement: S1 File — (Table A) Linear slopes and quadrics slopes with outcome values. (Table B) Distribution of individuals to different subgroups per movement behavior outcome expressed in percentages. (DOCX) [file pone.0229587.s001.docx]

**Supplementary materials - Movement behavior remains stable in stroke survivors within the first two months after returning home**

Roderick Wondergem^1,2,3^; Martijn F. Pisters^1,2,3^; Martijn W. Heijmans^4^; Eveline J.M. Wouters^3,5^; Rob A. de Bie^6^; Cindy Veenhof^1,2,7^; Johanna M.A. Visser-Meily^2,8^

^1^Center for Physical Therapy Research and Innovation in Primary Care, Julius Health Care Centers, Utrecht, The Netherlands; ^2^Department of Rehabilitation, Physical Therapy Science and Sport, Brain Center, University Medical Center Utrecht, University Utrecht, Utrecht, the Netherlands; ^3^Department of Health Innovations and Technology, Fontys University of Applied Sciences, Eindhoven, The Netherlands; ^4^Amsterdam UMC, location VU University Medical Center, Department of Epidemiology and Biostatistics, Amsterdam Public Health Research Institute, Amsterdam, The Netherlands; ^5^Tilburg University, School of Social and Behavioral Sciences, Department of Tranzo, Tilburg, The Netherlands; ^6^Maastricht University, Department of Epidemiology and Caphri Research School, Maastricht, The Netherlands; ^7^Expertise Center Healthy Urban Living, Research Group Innovation of Human Movement Care, University of Applied Sciences Utrecht, the Netherland; ^8^Center of Excellence for Rehabilitation Medicine, Brain Center, University Medical Center Utrecht and De Hoogstraat Rehabilitation, Utrecht, The Netherlands

Table A. Linear slopes and quadrics slopes with outcome values

| Linear slopes | | | | | | quadratic slopes | | | |  |
| --- | --- | --- | --- | --- | --- | --- | --- | --- | --- | --- |
|  |  | BIC | Entropy | BLRT | Subgroup size | BIC | Entropy | BLRT | Subgroup size | |
| SB | 1 | 2779.10 | 1.00 | n.a. | 140 | 2555.16 | 1.00 | n.a. | 140 | |
|  | 2 | 2472.68 | 0.89 | <0.01 | 89/51 | 2343.72 | 0.87 | <0.01 | 90/50 | |
|  | 3 | 2386.56 | 0.90 | <0.01 | 80/41/19 | 2291.20 | 0.93 | <0.01 | 87/52/1 | |
|  | 4 | 2278.08 | 0.93 | <0.01 | 79/41/19/1 | 2232.75 | 0.92 | <0.01 | 80/46/13/1 | |
|  | 5 | 2292.91 | 0.94 | 1.00 | 79/41/19/1/0 | 2201.08 | 0.90 | <0.01 | 71/38/18/12/1 | |
| LPA | 1 | 2630.71 | 1.00 | n.a. | 140 | 2372.47 | 1.00 | n.a. | 140 | |
|  | 2 | 2351.55 | 0.88 | <0.01 | 90/50 | 2192.88 | 0.82 | <0.01 | 92/48 | |
|  | 3 | 2188.71 | 0.94 | <0.01 | 83/56/1 | 2212.64 | 0.89 | 1.00 | 92/48/0 | |
|  | 4 | 2186.64 | 0.94 | 1.00 | 81/54/5/0 | 2104.06 | 0.92 | 1.00 | 78/60/2/0 | |
|  | 5 | 2082.87 | 0.94 | 1.00 | 70/59/10/1/0 | 2123.83 | 0.93 | 1.00 | 78/60/2/0/0 | |
| MVPA | 1 | 1091.74 | 1.00 | n.a. | 140 | 815.02 | 1.00 | n.a. | 140 | |
|  | 2 | 685.10 | 0.91 | <0.01 | 96/44 | 537.05 | 0.91 | <0.01 | 104/36 | |
|  | 3 | 504.37 | 0.90 | <0.01 | 59/57/24 | 442.71 | 0.88 | <0.01 | 77/48/15 | |
|  | 4 | 402.59 | 0.94 | <0.01 | 56/55/25/4 | 365.09 | 0.92 | <0.01 | 65/38/32/5 | |
|  | 5 | 729.58 | 0.96 | 1.00 | 96/44/0/0/0 | 366.68 | 0.91 | <0.01 | 61/38/29/7/5 | |
| mvpa bout | 1 | 401.82 | 1.00 | n.a. | 140 | 182.34 | 1.00 | n.a. | 140 | |
|  | 2 | 63.47 | 0.98 | <0.01 | 123/17 | -56.00 | 0.96 | <0.01 | 121/19 | |
|  | 3 | -83.89 | 0.93 | <0.01 | 89/42/9 | -143.52 | 0.96 | <0.01 | 4/26/110 | |
|  | 4 | -118.71 | 0.93 | <0.01 | 87/39/10/4 | -16.47 | 0.98 | 1.00 | 121/19 | |
|  | 5 | -112.30 | 0.87 | <0.01 | 62/39/17/13/9 | 3.30 | 0.98 | 1.00 | 121/19 | |
| median | 1 | 5573.46 | 1.00 | n.a. | 140 | 5406.47 | 1.00 | n.a. | 140 | |
|  | 2 | 5299.26 | 0.91 | <0.01 | 111/29 | 5210.83 | 0.94 | <0.01 | 118/22 | |
|  | 3 | 5151.92 | 0.91 | <0.01 | 74/52/14 | 5132.47 | 0.86 | <0.01 | 71/52/17 | |
|  | 4 | 5093.48 | 0.90 | <0.01 | 63/51/21/5 | 5087.31 | 0.90 | <0.01 | 73/50/16/1 | |
|  | 5 | 5086.20 | 0.88 | <0.01 | 60/45/20/10/5 | 5097.66 | 0.83 | 0.67 | 54/45/25/13/3 | |

BIC= Bayesian information criterion, BLRT= bootstrap likelihood ratio test, SB= sedentary behavior, LPA= light physical activity, MVPA= moderate to vigorous physical activity

Statistical considerations for finding the most appropriate model included a Bayesian information criterion (BIC), entropy values and the bootstrap likelihood ratio test (BLRT). The lower the BIC score, the better the fit of the model. When BLRT was significant (p<0.05), the trajectory with k-subgroups had a better fit than k-1 trajectory subgroups. The entropy statistic was used for the reliability of the subgroup trajectories. Entropy scores above 0.8 are preferred. When less than 5% of the sample was assigned to a subgroup trajectory, a k-1 subgroup trajectory was chosen in favor.

Table B. Distribution of individuals to different subgroups per movement behavior outcome expressed in percentages

|  |  | Sedentary | | LPA | | MVPA | | MVPA bouts ≥10 bouts | | Weighted median sedentary bout length | |
| --- | --- | --- | --- | --- | --- | --- | --- | --- | --- | --- | --- |
|  |  | Highly sedentary | Less sedentary | Non-movers | Movers | Inactive | Active | Inactive | Active | Prolongers | Intermediate  Interrupters |
| Sedentary | Highly sedentary |  |  | 53.6 | 10.7 | 35.7 | 28.6 | 38.6 | 25.7 | 10.0 | 54.3 |
|  | Less sedentary |  |  | 12.1 | 23.6 | 19.3 | 16.4 | 25.0 | 10.7 | 0.0 | 35.7 |
| LPA | Non-movers |  |  |  |  | 37.1 | 28.6 | 39.3 | 26.4 | 10.0 | 55.7 |
|  | Movers |  |  |  |  | 17.9 | 16.4 | 24.3 | 10.0 | 0.0 | 34.3 |
| MVPA | Inactive |  |  |  |  |  |  | 49.2 | 5.7 | 7.1 | 47.9 |
|  | active |  |  |  |  |  |  | 14.2 | 30.7 | 2.9 | 42.1 |
| MVPA bouts ≥10 bouts | Inactive |  |  |  |  |  |  |  |  | 5.7 | 57.9 |
|  | Active |  |  |  |  |  |  |  |  | 4.3 | 32.1 |
| Weighted median sedentary bout length | Prolongers |  |  |  |  |  |  |  |  |  |  |
|  | Intermediate interrupters |  |  |  |  |  |  |  |  |  |  |

LPA= Light physical activity, MVPA= Moderate to vigorous physical activity
