# Supplementary material for: Movement behavior remains stable in stroke survivors within the first two months after returning home
Source: PLoS One. 2020 Mar 26;15(3):e0229587. doi: 10.1371/journal.pone.0229587 (PMC7098564; doi:10.1371/journal.pone.0229587)
Supplement: Attachment — Submitted filename: 191114_Response_to_the_reviewers.docx [file pone.0229587.s002.docx]

**Response to the reviewers - PONE-D-19-23463**

Movement behavior remains stable in stroke survivors within the first two months after returning home
PLOS ONE

Dear Editor,

Thank you very much for sending us the reviewers’ comments on our manuscript entitled *“Movement behavior remains stable in stroke survivors within the first two months after returning home”* (**PONE-D-19-23463**).

The most important change we have made is the addition of characteristics associated with the different subgroups. The descriptions of the associated characteristics provide important information for clinicians to target those with an increased risk of sedentary behavior and low levels of physical activity.

Below, we have responded to each comment and explained our changes in the manuscript. Within the manuscript, changes are marked with strikethrough, and additions are highlighted.

We hope that you will consider our manuscript for publication in PLOS ONE.

Yours sincerely, on behalf of the authors,

Roderick Wondergem, MSc

**Editor’s comment:**
Apart from the comments made by the reviewers the discussion is lacking a comparison with "healthy" individuals. You state that "the majority of the stroke survivors are inactive and highly sedentary" while report that they spent 0.7 h daily on at least moderate intensity which is approximately equivalent to 42 minutes of MVPA daily. This is around twice the amount compared to a general population. Thus the conclusion seems not supported by your data.

**Authors’ response:**

Comparing general populations in Europe, the country with the highest proportion of people who regularly engage in physical activity is the Netherlands. Additionally, the Netherlands has the lowest proportion of those who never engage in physical activity(1). Although the overall average time spent engaged in moderate intensity physical activity is 0.7 hours daily, three MVPA trajectory subgroups were identified, showing that only 10.7% of participants had a “highly active” trajectory, and 34.3% had an “active” trajectory. The majority of the participants (55%) had an “inactive” MVPA trajectory (see table 3). The classification of MVPA in bouts ≥10 minutes slightly increased the percentage in the inactive MVPA trajectory subgroup. Based on these results, we concluded that the majority of stroke survivors are inactive. However, we agree with the editor that our statement needs to be adjusted based on the average for the total population. Additionally, we added a paragraph in the discussion section regarding the distribution.

**Authors’ action:**

We adjusted the sentences in the conclusion as follows:

*“The majority of the stroke survivors are highly sedentary, and a substantial proportion are inactive.”*

We added a paragraph in the discussion section:

*“On average, our sample showed SB results comparable to those of a Dutch older adult population* (2)*. In our sample, the majority of the people were highly sedentary, with sedentary periods exceeding 9.5 hours. The relationship between sedentary time and mortality was more pronounced when sedentary periods exceeded 9.5 hours* (3)*. Therefore, the reduction of SB should be a secondary prevention target for people with stroke. On average, our sample engaged in 42 minutes of MVPA per day, which is high. It is known that the Dutch population is more active than its European peers*(1)*. In other stroke survivors, the same amount of MVPA was found (44 minutes)*(4)*. Although, the average amount of MVPA was high, a substantial part of the population was found to be inactive, particularly in terms of MVPA accumulated in bouts ≥10 minutes.”*

**Editor’s comment:**
Also you should focus on the validity issues raised by the reviewers.

**Authors’ response:**

We focused on the validity issues raised by the reviewers. Our response can be found below Comment 2 of Reviewer 1.

Reviewer #1:

The authors of this manuscript tackle a highly important topic, that is changes in physical activity in the sub-acute phase after stroke. While numerous studies have demonstrated low levels of physical activity in the chronic phase after stroke little is known about the trajectory of physical activity over time. For that reason, as well as the large sample size and prolong follow-up of physical activity using accelerometers, this paper would make an important contribution to this field. There are some concerns though that I would like the authors to consider.

**Authors’ response:**

We would like to thank the reviewer for the comments. They gave us the opportunity to improve this paper. We respond on your comments below.

Major comments
**Reviewer’s comment:** 1. The term “movement behavior” is broad and vague. This paper has clearly investigated different intensity domains of physical activity – therefore the terminology should be changed from movement behavior to physical activity throughout the manuscript.

**Authors’ response:**

We used the term movement behavior, following the international consensus on terminology published by Tremblay et al. (5) in 2017, because we investigated both sedentary behavior and different intensities of physical activity. Within each 24-hour day, three behaviors are distinguished: sleeping behavior, sedentary behavior and physical activity (of different intensities: light, moderate and vigorous). We used the term movement behavior since it includes both sedentary behavior and physical activity. However, we agree with the reviewer about the need to clarify the term.

**Authors’ action:** In the abstract and introduction, we explained the term movement behavior, and we clarified it by changing it to “movement behavior during waking hours” since sleeping was not investigated.

Change in the title

*Movement behavior during waking hours remains stable in stroke survivors within the first two months after returning home*

Change in the abstract

*“The aim of this study is to investigate changes in movement behavior during waking hours and to identify subgroup trajectories in movement behaviors in first-time stroke patients in the first two months after discharge from the hospital to the home setting.”*

Furthermore, in the abstract, we changed “movement behavior” to “movement behavior outcomes”.

Changes in the manuscript

We changed “movement behaviors” to “movement behaviors during waking hours”. Examples:

*“Movement behaviors during waking hours include sedentary behavior (SB) and physical activity (PA)*(5)*. Within PA, the intensities of light physical activity (LPA) and moderate to vigorous physical activity (MVPA) can be distinguished.”*

And

*“The composition of movement behavior during waking hours (the relative amounts of SB, LPA and MVPA during waking hours), the continuity of SB (interrupted or prolonged SB), and the continuity of MVPA (bouts ≥10 minutes) are important modifiable risk factors to improve cardiovascular health.”*

**Reviewer’s comment:** 2. Please clarify the validity of the Activ8 accelerometer to detect sedentary, light and MVPA in people with stroke.

**Authors’ response:**

The Activ8 is a valid tool for measuring sedentary behavior and is able to distinguish between upright postures and sedentary behavior. Based on the results of previous validation studies that investigated similar stroke groups, we believe that the Activ8 is able to detect sedentary behavior. Specifically, research by Bussmann et al. has demonstrated 90.1% agreement between the output of Activ8 when worn in a pocket and a video analysis.(6). The Activ8 showed sufficient accuracy in detecting different classes of body postures and movements of people after a stroke during basic activities and daily life activities in a laboratory and/or at home (7). Furthermore, in our own validation study, we found ICCs ranging from 0.8 to 0.9 between the pocket-worn Activ8 and the elastic belt-fixed Movemonitor (which was used as a reference standard) (this study is currently under review)(8).

The Activ8 is a valid tool for measuring light physical activity since it is able to distinguish correctly between standing and sitting. Standing is not included in sedentary behavior but is considered light physical activity, and it can be detected correctly according to the study of Fanchamps et al. and our own validity study(7,8). Most importantly, Oomen et al. investigated the validity of the Activ8 regarding energy expenditures in a sample of healthy adults(9). The study investigated energy expenditures in walking (at different speeds) and running and found a correlation of 0.97. Based on these results, the distinction between light physical activity (walking) and moderate physical activity can be adequately made.

It is important to mention that Verschuren et al.(10) found that among people with stroke, energy expenditures during sedentary behavior do not exceed 1.5 MET. Additionally, walking at a slow pace has an energy expenditure below 3.0 MET and is therefore correctly classified as light physical activity. As shown in the study of Oomen et al., the Activ8 is able to distinguish between walking at a slow speed and at a faster speed. Additionally, our sample had minor stroke with a relatively high walking speed, which indicates generally normal physical abilities.

Additionally, in general, the Activ8 has specific characteristics that are important for detecting movement behaviors during waking hours and estimating energy expenditures. The thigh is a good place to wear an accelerometer for activity recognition and intensity levels. The Activ8 used an activity-specific estimation of energy expenditure based on an intensity factor(11,12). Activity-specific estimation methods have been found to perform better than count-based estimation methods and activity-specific estimation methods using a MET chart (13).

Therefore, we are convinced that the Activ8 is able to classify energy levels correctly.

**Authors’ action**

We added information to the text regarding the validation of the energy expenditure measurement:

*“The Activ8 has been validated in a community-living stroke population in terms of postures and in a healthy population in terms of energy expenditure* (7,9)*.”*

**Reviewer one had various questions about why we did not provide group characteristics. These questions are grouped below to improve readability of our response to the reviewer. Our response is below the reviewer’s comments.**

**Reviewer’s comment:** 3. The second aim; detecting possible subgroup trajectories within each outcome (i.e. different intensities of physical activity) using latent class growth analyses. To me, this section is statically driven and I would like the authors to justify this choice. For example, by explaining how detection of sub-groups following certain trajectory in physical activity would inform secondary prevention strategies post-stroke. Personally, I believe it is important to identify personal and functioning predictors of a “positive” vs “negative” (i.e. sedentary) physical activity trajectory post-stroke – and data from this trial seem to be able to contribute to this. Describing and contrasting the characteristics of stroke survivors following different trajectories could inform clinicians and researchers to target those of increased risk of sedentary and worsen cardio-vascular health. Taken together, to increase the influence of this paper, I think the rational and implication of the findings of the second aim should be clarified.

**Reviewer’s comment:** 5. As shown in table 1, functioning and stroke severity data was also collected in this study. Please justify why changes in these outcomes was not considered in relation to changes in physical activity.

**Reviewer’s comment:** 8. The introduction is overall well written, easy to follow, and introducing the reader to the research topic. My only comment is in line with my previous comment regarding aim 2, how would identification of certain subgroup trajectories of physical activity (without identifying who these individuals are) inform secondary stroke prevention?

**Authors’ response:**

First, our aim was to obtain better insight into the course of single-movement behavior outcomes and determine whether there are subgroups of patients with increases or decreases in one of these single aspects. Additionally, we expected trajectories of changes in movement behavior outcomes. Therefore, the method had to be data driven. We hypothesized that at least a subgroup of the population would improve on one of the single-movement behavior outcomes. This insight provides additional information to identify people with an unfavorable movement behavior. The identification of people with an unfavorable movement behavior is essential to including the right people in future secondary prevention interventions. Although we did identify subgroups, there were only small changes in the subgroup trajectories of sedentary behavior and MVPA. Based on the small changes and the identified subgroup trajectories, more research is needed to identify movement behavior patterns.

Before submitting our article, we had multiple discussions within our research group regarding whether the subgroup characteristics should be presented. In the end, we decided not to present these characteristics since only small changes were found in the subgroup trajectories. At the time, we thought that focusing only on the subgroup trajectories would improve readability and present a clear message. However, since both reviewers pointed out the absence of information about subgroup characteristics, we feel confident in the value of presenting the characteristics associated with the identified subgroups.

**Authors’ actions:**

We explained why better insight into the patterns is important, and we added the subgroup characteristics and, consequently, a third research question.

We changed the last paragraph of the introduction as follows:

*“Since stroke recovery is heterogeneous, and average group data assumes a one-size-fits-all principle, possible changes in movement behavior outcomes in terms of subgroup trajectories may be overlooked. Therefore, subgroup trajectories of change in movement behavior outcomes need to be investigated, since they are expected. To identify potential subgroup trajectories, data-driven analyses are needed. Latent class growth analysis is a method whereby participants are assumed to belong to a single class, but which class is not known*(14)*. This approach will extend our understanding of subgroup trajectories of change in movement behavior outcomes. Once these subgroup trajectories are known, associations need to be explored. Therefore, the aim of the current study is 1) to investigate changes in both the distribution (SB, LPA, and MVPA) and accumulation (bouts) of movement behavior during waking hours for the entire sample, and 2) to detect possible subgroup trajectories within each movement behavior outcome within the first two months after discharge from hospital care to the home setting in first-time stroke patients. Once these subgroup trajectories are known, 3) associated patient characteristics will be explored.”*

We added a third research aim in the introduction section:

*“Once these subgroup trajectories are known, associated patient characteristics will need to be explored.*

We also added research aim 3:

*3) to explore characteristics associated with identified subgroups.*

We added data analysis information to the method section:

*“If more than two subgroup trajectories were found based on the latent class growth analysis, trajectories were merged into two clinically relevant subgroups. To determine the characteristics associated with a single subgroup trajectory, logistic regression analyses were performed. Odds ratios were calculated to identify candidate factors using univariate analyses. The related variables were tested for multicollinearity (Pearson’s r < 0.70) and effect modification (variance inflation factor >4)* (15)*. Significantly associated characteristics (p<0.1) were entered into a multiple backward logistic regression analysis.”*

We added the results to the result section:

*“The ‘active’ and ‘highly active’ subgroup trajectories for both MVPA and MVPA spent in bouts ≥10 minutes were merged together since the participants in both subgroups were sufficiently active, as international guidelines recommend at least 150 minutes per week of accumulated moderate to vigorous physical activity (MVPA)* (16)*. Additionally, the ‘intermediate’ and ‘interrupters’ subgroup trajectories for the weighted median sedentary bout length were merged. Although there are no clear cut-off values available for the interruption of SB, interruption after thirty minutes has been found to have health benefits in people with stroke* (17,18)*. The distribution of individuals into the different subgroups is presented in supplementary materials table 2. The results show that the different movement behavior outcomes reveal distinct trajectories. For example, 53.6% of the population was highly sedentary and classified as nonmovers, and 35.7% was inactive and highly sedentary.*

*The results of the univariate analyses per movement behavior subgroup are presented in supplementary material table 3.*

*“The results of the multiple logistic regression analyses are presented in table 4. No associations were found regarding SB. Factors associated with nonmovers were living with another person and impaired cognitive function. Being male and younger and having fewer activity limitations were associated with both active groups (MVPA and MVPA spent in bouts ≥10 minutes). Living alone and being a community walker were only associated with the active MVPA group. Factors associated with prolongers were more severe stroke symptoms, cognitive impairment and not being a community walker.”*

We added the following information to the discussion section:

*“Remarkably, we found no patient characteristics that were associated with highly sedentary behavior. A recent study, which pooled data from nine studies identifying associations with sedentary time after stroke, found that sedentary time could not be explained by demographic or stroke-related variables*(19)*. It identified only slower walking speed as a significant factor associated with higher amounts of SB. In our sample, people were discharged immediately to the home-setting and had a relatively high walking speed, whereas the study of Hendrickx et al. included participants with greater diversity of walking speed. This could explain why walking speed was not identified as a factor associated with SB in our sample. Although living alone was associated with the total MVPA time, it was not associated with MVPA accumulated in bouts ≥10 minutes. It seems that people who live alone spend time in MVPA during their ADLs and devote less leisure time MVPA in such forms as exercise or sports. More severe stroke symptoms, cognitive impairment and not being a community walker were associated with prolongers in our study. These outcomes are in line with previous studies of people with stroke, although those studies found associations with walking speed, more severe stroke symptoms and self-reported ADLs and SBs* (19,20)*. The association between cognitive impairment and nonmovers and prolongers is interesting since no associations were found with total sedentary time or MVPA in our sample. A study including older adults found that higher amounts of SB were associated with lower cognitive function when MVPA was not taken into account; however, no association was found after adjustment for MVPA. This indicates the importance of investigating movement behavior patterns and not just single movement behavior outcomes. Additionally, it could be that the health benefits of enough MVPA are counterbalanced by high amounts of SB.”*

**Reviewer’s comment:** 4. Does the results for the physical activity outcomes (sedentary, light and MVPA) reveal unique trajectories or is the results demonstrating a single trajectory of sedentary behavior post-stroke (see comment 11 below). Please clarify.

**Authors’ response:** We would like to thank the reviewer for this interesting question. The identified trajectories are indeed unique, since individuals were distributed differently among the movement behavior outcomes. For example, 53.6 percent of the population was highly sedentary and considered nonmovers; however, 35.7 percent was considered inactive and highly sedentary. Therefore, in the discussion section, we recommend the identification of unique movement behavior patterns.

**Authors’ action:** We have added the distribution of individuals among different subgroups according to movement behavior outcome in the supplementary information.

Abstract
**Reviewer’s comment:** 6. Results; I would suggest to present the percentage of total wear time for each intensity (sedentary, light and MVPA) as it provides an informative summary of the distribution between intensity categories.

**Authors’ response:** We agree with the reviewer that percentages provide additional information. Therefore, we have added this information in the abstract.

**Reviewer’s comment:** 7. Result (first sentence): It is unclear whether this sentence refer to the entire time period for data collection or a certain time period.

**Authors’ response:** We have clarified that this sentence refers to the mean values for the first week.

Method
Recruitment and inclusion criteria are clearly stated.

**Reviewer’s comment:** 9. Performance based tests of balance and gait were undertaken in the participants home. Berg Balance Scale and 5MWT requires some space to be performed, was it possible to perform these tests in all subject’s home in a standardized manner?

**Authors’ response:** Both tests were performed in a standardized manner. It was possible to perform the 5 MWT in all homes. Additional equipment for the Berg Balance Scale was brought to the participants’ homes to ensure that the test was performed in a standardized manner.

**Reviewer’s comment:** 10. In table 1, data on the provision of physiotherapy care is provided, but I cannot find any information of these variables in the method section. Please a description of these variables including the nature, timing and definitions of these services as they could differ between health care systems.

**Authors’ response:** Stroke services are a form of integrated care that was established during the last decade. The aim of stroke services is to improve health outcomes and processes of care by connecting the acute, rehabilitative, and chronic phases of stroke care(21,22). In a typical stroke service, the hospital, rehabilitation center, nursing home (geriatric rehabilitation) and primary care physiotherapy are represented.

**Authors’ action:**

We added in the text:

*“Stroke services are a form of integrated care that was established during the last decade. The aim of stroke services is to improve health outcomes and processes of care by connecting the acute, rehabilitative, and chronic phases of stroke care(21,22). In a typical Dutch stroke service, the hospital, rehabilitation center, nursing home (geriatric rehabilitation) and primary care physiotherapy are represented. Information about physiotherapy care was obtained from medical records and verified by the participant. Three options were possible: no physiotherapy care, primary physiotherapy care and outpatient multidisciplinary rehabilitation care that included physiotherapy.”*

Results
**Reviewer’s comment:** 11. Results are overall clearly presented. As pointed out in my previous comment, describing the characteristics of the stroke survivors following different trajectories would be informative. Also, to what extent does the trajectories overlap between the different PA outcomes? For instance, 90 participants were defined as highly sedentary, 92 as non-movers according to light PA and 89 as inactive according to MVPA. The weighted median sedentary bout length also adds up to about 90 if combining the “prolongers” (n=14) and intermediate groups (n=74). Are these PA outcomes revealing a single trajectory of sedentary behavior post-stroke occurring in approximately 65% of this sample? Or do the different PA intensities reveal unique patterns? I am curious about the authors thoughts on this potential overlap and it would be very interesting if data supporting or rejecting such overlap could be presented.

**Authors’ response:** We would like to thank the reviewer for this interesting question. This comment is in line with comment 4. We have provided data in the supplementary materials that rejects the hypothesis that the trajectories are overlapping.

**Authors’ action:** In addition to the information in the supplementary materials, we have added the following sentences to the beginning of paragraph three in the discussion section:

*“Trajectories of single movement behavior outcomes overlap; however, they are largely unique. For example, 54% of the people who were highly sedentary were nonmovers, but only 36% of the highly sedentary people were inactive.”*

**Reviewer’s comment:** 12. Please add information on time since stroke when the assessment of physical activity started – such data would allow comparison with similar longitudinal studies.

**Authors’ action:**

We have added the time since stroke in table 1 (19.6 days, with a standard deviation of 5.6).

**Reviewer’s comment:** 13. Figure 1 is blurry – please improve the quality. In addition, I would suggest adding 95% confidence intervals to the estimates.

**Authors’ response and action:** We agree with the reviewer that the figure is blurry. The figures were automatically generated by Mplus and are not of satisfactory quality. Therefore, we have included higher quality figures that include 95% CIs.

Discussion
Relevant and clear – linked and contrasted to previous body of research.

**Reviewer’s comment:** 14. First sentence (first paragraph): it is stated that the assessment took place “in the first 2 months after discharge” indicating a period of data collection of 10 weeks. Please rephrase.

**Authors’ response and action:** The mean time after stroke was 20 days when data collection started. In total, 35 days were measured. Therefore, we rephrased the statement as “*within the first 2 months after discharge”* in the title, abstract, research question 2 and discussion section. We have added the following sentence to the discussion section:

*This study investigated changes in movement behavior outcomes and possible subgroup trajectories in 140 participants within the first two months, by objectively measuring continuously for five weeks, after discharge from the hospital to the home setting in people with a first-ever stroke.*

**Reviewer’s comment:** 15. The authors might need to revise the reasoning in the discussion regarding the unique trajectories for different intensity outcomes; e.g. “distinctive subgroup trajectories were found for each movement behavior outcome”, depending on whether they are unique patterns or not.

**Authors’ response:** Since the hypothesis of overlapping trajectories was rejected, we did not revise these sentences.

List of abbreviations
**Reviewer’s comment:** 16. As a reader, it is rather demanding to keep track on all the short-forms used in this manuscript (n=16). I would recommend to only use short-forms of the most frequently used terms.

**Authors’ action:** We have left 5 abbreviations.

Reviewer #2: This is an interesting study with a relatively large n that examines the time course of activity levels during the first 2 months post stroke

Major:
**We separated the comment below in three comments.**

**Reviewer’s comment 1a:**

Why was activity measured only during the time frame from 3 weeks to 2 months post stroke?

Recovery post stroke is still likely happening past this time point.

**Authors’ response:**

Since longitudinal studies did not find changes in movement behavior outcomes up to twelve months after stroke on group level (4,23,24), we hypothesized that a decrease in total sedentary time, increased interruption of SB, and increases in LPA and MVPA would occur during the initial period after discharge. These outcomes were expected because most functional recovery occurs within the first few weeks after stroke (25), and most stroke survivors still receive professional support or rehabilitation during that period (26). Moreover, during those initial weeks, health care professionals provide information regarding modifiable risk factors, including movement behaviors (27). Additionally, it was expected that during the period shortly after stroke, survivors are especially motivated to improve their lifestyle to prevent recurrent events (28) and establish movement behavior habits. Based on the results, we had to conclude that movement behavior remains stable.

**Reviewer’s comment 1b:**

Also, were the participants still receiving home or out patient rehabilitation during this time (I see a brief amount of info on this in table 1, but there is no mention of it anywhere else, please elaborate on this, also was there any difference in activity levels based on receiving or not receiving rehab?). Both of these factors could greatly influence activity levels (time in sitting/lying, etc)?

**Authors’ response:**

Since 46,4% of the sample received rehabilitation care (primary physiotherapy or outpatient rehabilitation), we thought that this group at least would show an increase in MVPA. However, we did not observe such a trajectory, nor did we observe an association between PT and MVPA.

**Reviewer’s comment 1c:**

Related to this please provide information on exactly when post stroke the participants started wearing the devices and stopped. Just a blanket statement of 3 weeks post discharge from the hospital is not sufficient. This is important information in order to interpret the activity levels.

**Authors’ response:**

We added time since stroke to table one.

**Reviewer’s comment:** What are the clinical characteristics of the different subgroups (i.e. GS, BBS, etc.), are there any differences in these between the groups?

**This comment is in line with the comments of reviewer one. Therefore, we have copied our response and actions here.**

**Authors’ response:**

This research was conducted to investigate the course of single movement behaviors to determine whether subgroups would show improvements or decreases in one of the single aspects. Additionally, we expected trajectories of changes in movement behavior outcomes. Therefore, the method had to be data driven. We hypothesized that at least a subgroup of the population would improve on one of the single-movement behavior outcomes. This insight provides additional information to identify people with an unfavorable movement behavior. The identification of people with an unfavorable movement behavior is essential to including the right people in future secondary prevention interventions. Although we did identify subgroups, there were only small changes in the subgroup trajectories of sedentary behavior and MVPA. Based on the small changes and the identified subgroup trajectories, more research is needed to identify movement behavior patterns.

Before submitting our article, we had multiple discussions within our research group regarding whether the subgroup characteristics should be presented. In the end, we decided not to present these characteristics since only small changes were found in the subgroup trajectories. At the time, we thought that focusing only on the subgroup trajectories would improve readability and present a clear message. However, since both reviewers pointed out the absence of information about subgroup characteristics, we feel confident in the value of presenting the characteristics associated with the identified subgroups.

**Authors’ actions:**

We added the third research question to the introduction section with a clarification:

*“Once these subgroup trajectories are known, their associations need to be explored to ensure that the right patients are included in future interventions.”*

We also added question 3 to the list of research questions:

*3) to explore the characteristics associated with the identified subgroups.*

We added information about the data analyses to the method section:

*“If more than two subgroup trajectories were found based on the latent class growth analysis, trajectories were merged into two clinically relevant subgroups. To determine the characteristics associated with a single subgroup trajectory, logistic regression analyses were performed. Odds ratios were calculated to identify candidate factors using univariate analyses. The related variables were tested for multicollinearity (Pearson’s r < 0.70) and effect modification (variance inflation factor >4)* (15)*. Significantly associated characteristics (p<0.1) were entered into a multiple backward logistic regression analysis.”*

We added the following results to the results section:

*“The ‘active’ and ‘highly active’ subgroup trajectories for both MVPA and MVPA spent in bouts ≥10 minutes were merged together since the participants in both subgroups were sufficiently active, as international guidelines recommend at least 150 minutes per week of accumulated moderate to vigorous physical activity (MVPA)* (16)*. Additionally, the ‘intermediate’ and ‘interrupters’ subgroup trajectories for the weighted median sedentary bout length were merged. Although there are no clear cut-off values available for the interruption of SB, interruption after thirty minutes has been found to have health benefits in people with stroke* (17,18)*. The distribution of individuals into the different subgroups is presented in supplementary materials table 2. The results show that the different movement behavior outcomes reveal distinct trajectories. For example, 53.6% of the population was highly sedentary and classified as nonmovers, and 35.7% was inactive and highly sedentary.*

*The results of the univariate analyses per movement behavior subgroup are presented in supplementary material table 3.*

*“The results of the multiple logistic regression analyses are presented in table 4. No associations were found regarding SB. Factors associated with nonmovers were living with another person and impaired cognitive function. Being male and younger and having fewer activity limitations were associated with both active groups (MVPA and MVPA spent in bouts ≥10 minutes). Living alone and being a community walker were only associated with the active MVPA group. Factors associated with prolongers were more severe stroke symptoms, cognitive impairment and not being a community walker.”*

We added the following to the discussion section:

*“Remarkably, we found no patient characteristics that were associated with highly sedentary behavior. A recent study, which pooled data from nine studies identifying associations with sedentary time after stroke, found that sedentary time could not be explained by demographic or stroke-related variables*(19)*. It identified only slower walking speed as a significant factor associated with higher amounts of SB. In our sample, people were discharged immediately to the home-setting and had a relatively high walking speed, whereas the study of Hendrickx et al. included participants with greater diversity of walking speed. This could explain why walking speed was not identified as a factor associated with SB in our sample. Although living alone was associated with the total MVPA time, it was not associated with MVPA accumulated in bouts ≥10 minutes. It seems that people who live alone spend time in MVPA during their ADLs and devote less leisure time MVPA in such forms as exercise or sports. More severe stroke symptoms, cognitive impairment and not being a community walker were associated with prolongers in our study. These outcomes are in line with previous studies of people with stroke, although those studies found associations with walking speed, more severe stroke symptoms and self-reported ADLs and SBs* (19,20)*. The association between cognitive impairment and nonmovers and prolongers is interesting since no associations were found with total sedentary time or MVPA in our sample. A study including older adults found that higher amounts of SB were associated with lower cognitive function when MVPA was not taken into account; however, no association was found after adjustment for MVPA. This indicates the importance of investigating movement behavior patterns and not just single movement behavior outcomes. Additionally, it could be that the health benefits of enough MVPA are counterbalanced by high amounts of SB.”*

*.*

**Reviewer’s comment:** This was a very high functioning group in regards to mobility as evidenced by the mean gait speed of 1.03 m/s, this should be at least discussed as a limitation.

**Authors’ response:** Although it is not truly a limitation of the study since we only wanted to include people who were discharged immediately to the home setting, we agree with the reviewer that it is important to mention that this population had mainly minor stroke symptoms.

**Authors’ action:**

We have added the following sentences to the limitation paragraph of the discussion section:

*“Last, our study included only participants who were directly discharged to the home setting. Since the majority of this population had minor stroke symptoms, the results are not generalizable to a more severe stroke population that received inpatient rehabilitation first. However, our findings emphasize the importance of movement behavior changes since our sample had less severe stroke symptoms but still presented high levels of SB and low levels of MVPA.”*

**Reviewer’s comment:** The Activ8 device has not been validated to identify energy expenditure in people with stroke, the article cited demonstrates its ability to identify postures (lying/sitting, standing, and walking. The authors use the outcomes time in SB, LP, and MVPA with associated METs. This is misleading as the device has not been validated to do this in people with stroke. Additionally, the method used by the authors to secure the device is not consistent with the study cited. To be more accurate the authors should use time spent in the different postures as their outcomes, not in SB, LP, MVPA.

**Since this is the same comment that reviewer 1 made, we have copied our response.**

**Authors’ response:**

In general, the Activ8 has specific characteristics that are important for detecting movement behaviors during waking hours and estimating energy expenditures. The thigh is a good place to wear an accelerometer for activity recognition and intensity levels. The Activ8 used an activity-specific estimation of energy expenditure based on an intensity factor(11,12). Activity-specific estimation methods have been found to perform better than count-based estimation methods and activity-specific estimation methods using a MET chart (13).

The Activ8 is a valid tool for measuring sedentary behavior and is able to distinguish between upright postures and sedentary behavior. Based on the results of previous validation studies that investigated similar stroke groups, we believe that the Activ8 is able to detect sedentary behavior. Specifically, research by Bussmann et al. has demonstrated 90.1% agreement between the output of Activ8 when worn in a pocket and a video analysis(6). The Activ8 showed sufficient accuracy in detecting different classes of body postures and movements of people after a stroke during basic activities and daily life activities in a laboratory and/or at home (7). Furthermore, in our own validation study, we found ICCs ranging from 0.8 to 0.9 between the pocket-worn Activ8 and the elastic belt-fixed Movemonitor (which was used as a reference standard) (this study is currently under review)(8).

The Activ8 is a valid tool for measuring light physical activity since it is able to distinguish correctly between standing and sitting. Standing is not included in sedentary behavior but is considered light physical activity, and it can be detected correctly according to the study of Fanchamps et al. and our own validity study(7,8). Most importantly, Oomen et al. investigated the validity of the Activ8 regarding energy expenditures in a sample of healthy adults(9). The study investigated energy expenditures in walking (at different speeds) and running and found a correlation of 0.97. Based on these results, the distinction between light physical activity (walking) and moderate physical activity can be adequately made.

It is important to mention that Verschuren et al.(10) found that among people with stroke, energy expenditures during sedentary behavior do not exceed 1.5 MET. Additionally, walking at a slow pace has an energy expenditure below 3.0 MET and is therefore correctly classified as light physical activity. As shown in the study of Oomen et al., the Activ8 is able to distinguish between walking at a slow speed and at a faster speed. Additionally, our sample had minor stroke with a relatively high walking speed, which indicates generally normal physical abilities.

Therefore, we are convinced that the Activ8 is able to classify the energy levels correctly.

**Reviewer’s comment:** The authors state that the small decrease in SB and increase in LPA are likely not clinically important. Although they are likely correct in this assessment they should provide some evidence of this.

**Authors’ response:** Although we wrote that these changes are “potential clinically important”, we have added further information since recently, Ekelund et al. found that all intensities of physical activity and less sedentary time are associated with a substantially reduced risk of death in a dose-response manner. Therefore, we adjusted the first sentences of this paragraph.

**Authors’ action:**

The first sentences of the second paragraph in the discussion section have been adjusted:

*“Although a significant decrease in SB and an increase in time spent in LPA were found within the first two months after discharge, the changes were small. However, it was recently found that higher levels of physical activity, including light physical activity, and less time spent in SB reduce the risk of premature death in a dose-response manner* (3)*. Therefore, even this small change in LPA and SB is considered relevant. Nevertheless, the absolute amount of SB was still high.”*

Additionally, we added information about the subgroup trajectory changes to the end of the third paragraph:

*“Differences in the changes among the subgroup trajectories were found. Participants in the highly sedentary subgroup trajectory decreased their amount of sedentary time, and those in the inactive group increased their MVPA time. Both changes, in theory, can reduce the risk of premature death, although the changes are small* (3)*.”*

Minor:
**Reviewer’s comment:** The statement associated with reference 4 in the first paragraph is misleading. Only systolic BP was found to impacted by sedentary lifestyle intervention; cardiovascular event rate mortality, diastolic blood pressure, or total cholesterol were not. Please revise.

**Authors’ response:** We wrote *“secondary lifestyle interventions are important and demonstrated to be effective in reducing cardiovascular risk after stroke* (29)*”* and did not mention cardiovascular event rate mortality, diastolic blood pressure, or total cholesterol. Because the reduction of systolic blood pressure is one of the strongest risk factors for both first and recurrent stroke(30), we do not think this statement is misleading. Nevertheless, we have adjusted the sentence to make it as clear as possible.

**Authors’ action:**

The following sentence was changed:

*“secondary lifestyle interventions are important and demonstrated to be effective in reducing systolic blood pressure, one of the strongest risk factors for both first and recurrent stroke”*

**Reviewer’s comment:** The statement related to reference 14 in the second paragraph is also misleading. The article cited is a study protocol, they did not determine that interruption of sedentary bouts with activity reduces BP.

**Authors’ response:**

We agree with the reviewer that this reference was incorrect since we referred to the study outcome and not its protocol. Therefore, we changed the reference as follows:

*English et al. Frequent, short bouts of light-intensity exercises while standing decreases systolic blood pressure: Breaking Up Sitting Time after Stroke (BUST-Stroke) trial.*(31)

**Reviewer’s comment:** 5th paragraph of the discussion, the point about the current approach is not clear and misleading. This was not an intervention study so there was no “approach” to lessen SB and increase activity levels. Also it is not clear how many participants were receiving rehabilitation services.

**Authors’ response:**
In our opinion, we did not mislead the reader. Perhaps the explanation was not clear; therefore we adjusted this paragraph by explaining that the current approach to improving capabilities will not automatically improve daily physical activity. Additionally, 54% of the population did not receive physiotherapy care, and a substantial proportion of this group could benefit from a focus on improving daily physical activity.

**Authors’ action:**
We adjusted the 5^th^ paragraph:

*“Although we expected to observe more changes in movement behavior outcomes based on the efforts of health care professionals, the willingness to change because of having experienced a stroke and the fact that recovery was feasible at the time of the study, only small changes in movement behavior outcomes occurred. In this sample, 46% of the population received physiotherapy care. In general, physiotherapy care focuses on regaining physical function and improving physical fitness*(32)*. However, improvements in capabilities due to functional recovery will automatically improve ADLs, but will not automatically improve daily physical activity* (33) *or reduce SB. Additionally, as a general practice, all people with stroke in the Netherlands are included in primary care programs. However, within these programs, there is limited attention to secondary prevention after stroke, especially physical activity* (34,35)*. Furthermore, changing movement behavior is a complex process and cannot be triggered by merely providing information* (36,37)*. Therefore, specific interventions are needed to improve daily physical activity and decrease sedentary time. Particularly since the majority of our sample was sedentary and a substantial part was inactive, improving movement behavior is important and needs to be targeted to counterbalance increased cardiovascular risks. Additionally, the participants in this study had relatively minor stroke symptoms but nonetheless were highly sedentary, and a substantial proportion of the sample was inactive. Although it is possible to modify daily physical activity and SB, it is not possible at present to suggest the superiority of a particular intervention approach* (38)*.”*

1.	Bennie JA, Wiesner GH, van Uffelen JGZ, Harvey JT, Craike MJ, Biddle SJHH, et al. Special Eurobarometer 472 Summary Sport and physical activity Fieldwork December 2017 Publication Survey requested by the European Commission , Special Eurobarometer 472 Summary. Int J Behav Nutr Phys Act. 2018;

2.	van Ballegooijen AJ, van der Ploeg HP, Visser M. Daily sedentary time and physical activity as assessed by accelerometry and their correlates in older adults. Eur Rev Aging Phys Act. 2019;16:3.

3.	Ekelund U, Tarp J, Steene-Johannessen J, Hansen BH, Jefferis B, Fagerland MW, et al. Dose-response associations between accelerometry measured physical activity and sedentary time and all cause mortality: systematic review and harmonised meta-analysis. BMJ. 2019;

4.	Baert I, Daly D, Dejaeger E, Vanroy C, Vanlandewijck Y, Feys H. Evolution of cardiorespiratory fitness after stroke: A 1-year follow-up study. influence of prestroke patients’ characteristics and stroke-related factors. Arch Phys Med Rehabil. 2012;93:669–76.

5.	Tremblay MS, Aubert S, Barnes JD, Saunders TJ, Carson V, Latimer-Cheung AE, et al. Sedentary Behavior Research Network (SBRN) - Terminology Consensus Project process and outcome. Int J Behav Nutr Phys Act. 2017;14.

6.	Bussmann JBJ. Validation of the Active8 Activity Monitor: detection of body postures and movements. Erasmus. 2013;

7.	Fanchamps MHJ, Horemans HLD, Ribbers GM, Stam HJ, Bussmann JBJ. The accuracy of the detection of body postures and movements using a physical activity monitor in people after a stroke. Sensors (Switzerland). 2018;18.

8.	Wondergem R, Kroes S, Wouters EJ, Janssen D, de Bie RA, Veenhof C, et al. Criterion and structural validity of the Activ8 accelerometer in community walking people with stroke. submitted.

9.	Oomen J, Arts D, Sperling M, Vos S. A stepwise science-industry collaboration to optimize the calculation of energy expenditure during walking and running with a consumer-based activity device. Technol Soc. 2019;

10.	Verschuren O, de Haan F, Mead G, Fengler B, Visser-Meily A. Characterizing Energy Expenditure During Sedentary Behavior After Stroke. Arch Phys Med Rehabil. 2016;97:232–7.

11.	H.K. Montoye A, M. Pivarnik J, M. Mudd L, Biswas S, A. Pfeiffer K. Validation and Comparison of Accelerometers Worn on the Hip, Thigh, and Wrists for Measuring Physical Activity and Sedentary Behavior. AIMS Public Heal. 2016;

12.	Cleland I, Kikhia B, Nugent C, Boytsov A, Hallberg J, Synnes K, et al. Optimal placement of accelerometers for the detection of everyday activities. Sensors (Basel). 2013;

13.	Altini M, Penders J, Vullers R, Amft O. Estimating energy expenditure using body-worn accelerometers: A comparison of methods, sensors number and positioning. IEEE J Biomed Heal Informatics. 2015;

14.	Jung T, Wickrama KAS. An Introduction to Latent Class Growth Analysis and Growth Mixture Modeling. Soc Personal Psychol Compass. 2008;2:302–17.

15.	Miles JN V., Shevlin ME. Applying regression and correlation: a guide for students and researchers. Sage Publications; 2001. 253 p.

16.	WHO. Global recommendations on physical activity for health. Journal of Chemical Information and Modeling. 2010.

17.	K. Lewis L, Hunt T, T. Williams M, English C, S. Olds T. Sedentary Behavior in People with and without a Chronic Health Condition: How Much, What and When? AIMS Public Heal. 2016;3:503–19.

18.	English C, Janssen H, Crowfoot G, Callister R, Dunn A, Mackie P, et al. Breaking up sitting time after stroke (BUST-stroke). Int J Stroke. 2018;

19.	Hendrickx W, Riveros C, Askim T, Bussmann JBJ, Callisaya ML, Chastin SFM, et al. Identifying factors associated with sedentary time after stroke. Secondary analysis of pooled data from nine primary studies. Top Stroke Rehabil. 2019;

20.	Tieges Z, Mead G, Allerhand M, Duncan F, van Wijck F, Fitzsimons C, et al. Sedentary behaviour in the first year after stroke: a longitudinal cohort study with objective measures. Arch Phys Med Rehabil. 2014;

21.	Minkman MMN, Schouten LMT, Huijsman R, Van Splunteren PT. Integrated care for patients with a stroke in the Netherlands: results and experiences from a national Breakthrough Collaborative Improvement project. Int J Integr Care. 2005;

22.	Rosendal H, Wolters CAM, Beusmans GHMI, De Witte LP, Boiten J, Crebolder HFJM. Stroke service in the Netherlands: an exploratory study on effectiveness, patient satisfaction and utilisation of healthcare. Int J Integr Care. 2002;

23.	Tieges Z, Mead G, Allerhand M, Duncan F, van Wijck F, Fitzsimons C, et al. Sedentary behavior in the first year after stroke: a longitudinal cohort study with objective measures. Arch Phys Med Rehabil. 2015;96:15–23.

24.	English C, Manns PJ, Tucak C, Bernhardt J. Physical activity and sedentary behaviors in people with stroke living in the community: a systematic review. Phys Ther. 2014;94:185–96.

25.	Langhorne P, Bernhardt J, Kwakkel G. Stroke rehabilitation. Lancet. 2011;377:1693–702.

26.	Marzolini S, Danells C, Oh PI, Jagroop D, Brooks D. Feasibility and Effects of Cardiac Rehabilitation for Individuals after Transient Ischemic Attack. J Stroke Cerebrovasc Dis. 2016;

27.	Sakakibara BM, Kim AJ, Eng JJ. A Systematic Review and Meta-Analysis on Self-Management for Improving Risk Factor Control in Stroke Patients. Int J Behav Med. 2017;

28.	Salinas J, Schwamm LH. Behavioral Interventions for Stroke Prevention: The Need for a New Conceptual Model. Stroke. 2017;48:1706–14.

29.	Deijle IA, Van Schaik SM, Van Wegen EEH, Weinstein HC, Kwakkel G, Van Den Berg-Vos RM. Lifestyle interventions to prevent cardiovascular events after stroke and transient ischemic attack. Stroke. 2017.

30.	Feigin VL, Roth GA, Naghavi M, Parmar P, Krishnamurthi R, Chugh S, et al. Global burden of stroke and risk factors in 188 countries, during 1990–2013: a systematic analysis for the Global Burden of Disease Study 2013. Lancet Neurol. 2016;

31.	English C, Janssen H, Crowfoot G, Bourne J, Callister R, Dunn A, et al. Frequent, short bouts of light-intensity exercises while standing decreases systolic blood pressure: Breaking Up Sitting Time after Stroke (BUST-Stroke) trial. Int J Stroke. 2018;

32.	Veerbeek JM, van Wegen EEH, van Peppen RPS, Hendriks HJM, Rietberg MB, van der Wees PJ, et al. KNGF-richtlijn Beroerte Verantwoording en toelichting. KNGF. 2014;121.

33.	Saunders DH, Greig CA, Mead GE. Physical activity and exercise after stroke: review of multiple meaningful benefits. Stroke. 2014;45:3742–7.

34.	Pedersen RA, Petursson H, Hetlevik I. Stroke follow-up in primary care: a prospective cohort study on guideline adherence. BMC Fam Pract. 2018;19:179.

35.	Olaiya MT, Cadilhac DA, Kim J, Nelson MR, Srikanth VK, Andrew NE, et al. Long-term unmet needs and associated factors in stroke or TIA survivors. Neurology. 2017;

36.	Prior PL, Suskin N. Exercise for stroke prevention. Vol. 3, Stroke and Vascular Neurology. 2018. p. 59–68.

37.	Gordon NF, Gulanick M, Costa F, Fletcher G, Franklin BA, Roth EJ, et al. Physical Activity and Exercise Recommendations for Stroke Survivors: An American Heart Association Scientific Statement From the Council on Clinical Cardiology, Subcommittee on Exercise, Cardiac Rehabilitation, and Prevention; the Council on Cardiovascular Nursing; the Council on Nutrition, Physical Activity, and Metabolism; and the Stroke Council. Circulation. 2004;109:2031–41.

38.	Kringle EA, Barone Gibbs B, Campbell G, McCue M, Terhorst L, Kersey J, et al. Influence of Interventions on Daily Physical Activity and Sedentary Behavior after Stroke: A Systematic Review. PM&R. 2019;pmrj.12222.
