# Supplementary material for: Movement behavior remains stable in stroke survivors within the first two months after returning home
Source: PLoS One. 2020 Mar 26;15(3):e0229587. doi: 10.1371/journal.pone.0229587 (PMC7098564; doi:10.1371/journal.pone.0229587)
Supplement: Attachment — Submitted filename: 200205 Response_.docx [file pone.0229587.s003.docx]

**Response to the reviewers - PONE-D-19-23463**

Movement behavior remains stable in stroke survivors within the first two months after returning home
PLOS ONE

Dear Editor,

Thank you very much for sending us the reviewers’ comments on our manuscript entitled *“Movement behavior remains stable in stroke survivors within the first two months after returning home”* (**PONE-D-19-23463**).

Below, we have responded to each comment and explained our changes in the manuscript. Within the manuscript, changes are marked with strikethrough, and additions are highlighted.

We hope that you will consider our manuscript for publication in PLOS ONE.

Yours sincerely, on behalf of the authors,

Roderick Wondergem, MSc

Reviewer #1: The authors have done an excellent job responding to the issues raised and clarifying these in the manuscript. Except for a minor issue with regards to Table 4, I have no further comments or issues that I want to the authors to address.

**Reviewer’s comment:** Table 4 reports the result for the multivariate logistic regression -I would suggest to include the results for the univariate logistic regression as a separate table as well. Also, all independent variables need to be understandable from reading only the table itself (without looping back to the method section) – please clarify the cut-points used for each nominal variables (i.e. similar to Table 1).

**Authors’ response and action:** We agree with reviewer that it will be more easy to interpret table 4 if the requested information is given. In addition the univariate logistic regression analyses are moved from the supplementary materials to the manuscript.

Reviewer #3

**Reviewer’s comment:** The topic of this work is major in the management of stroke patients in the field of physical activity. Evaluating changes in physical activity levels and sedentary behaviour in the home is central to identifying the determinants of these changes in order to propose effective actions. The authors do not elaborate much on this in their introduction and that is a pity.It is worth noting the authors' effort to have evaluated the participants in such a complete manner and to have carried out such a consistent follow-up on such a large group of patients.
The major limitation of this work is the lack of validation of activ8 for the estimation of energy expenditure or METs in stroke subjects. This is a major limitation because the entire classification process of the study concerns the quality of the device to differentiate the different categories of physical activity intensity performed by the individual stroke at home. Despite a quality work performed by Fanchamps et al showing a validity of physical activity detection, the transposition of METs on activities identified by Activ8 from healthy subject populations is inadequate cf Serra et al 2016, Compagnat et al 2018. In addition, this device uses a uniaxial accelerometer with an algorithm developed on a healthy subject. However, we now know that estimates of this type of device are not reliable in stroke subjects for the same reasons explained below. It therefore seems necessary to explore the validity of a device that has not been validated before being used in clinical research.
If the reader accepts this uncertainty I have developed some remarks about the manuscript.

**Authors’ response:**
We would like to thank the author for the compliments and agree that optimal distribution of physical activity and sedentary behavior is of high interested for people with stroke with regards to secondary prevention. We disagree however on the suggested major limitations of our work regarding the validation of the Activ8. First of all, the Activ8 is a tight worn triaxial accelerometer and not an uniaxial accelerometer as mentioned by reviewer 3. Below we will summarize earlier research that demonstrates acceptable accuracy in community dwelling and walking (FAC≥3) stroke survivors. In addition to the accuracy we have chosen the Activ8 because its user-friendly, easy to wear and able to monitor continuously up to thirty days without charging. These aspects have contributed to the wearing compliance.

The activ8 is a valid tool to measure sedentary behavior and is able to distinguish between upright postures and sedentary behavior. Results of previous validation studies, within a similar stroke groups, it can be concluded that the Activ8 is able to detected sedentary behavior accurately. Namely, research by Bussmann et al. has demonstrated 90% agreement between Activ8 output, worn in the pocket, and video analysis was 90.1% (1). The Activ8 showed sufficient accuracy in detecting different classes of body postures and movements of people after a stroke during basic activities and daily-life activities in a laboratory and/or at home (2). Furthermore, in our own validation study we found ICC’s ranging between 0.8 and 0.9 between the pocket worn Activ8 and the elastic belt fixed Movemonitor (which was used as a reference standard) (this study is currently under review)(3). The results are comparable to a more commonly used device, the Activpal(4,5).

The Activ8 is a valid tool to measure light physical activity since it is able to distinguish correctly between standing and sitting. Since standing is not included in sedentary behavior but in light physical activity it can be detected correctly according to the study of Fanchamps et al. and our own validity study (2,3). Most important Oomen et al investigated the validity of the Activ8 regarding energy expenditures in a sample of healthy adults(6). The study investigated energy expenditures in walking (at different speeds) and running and found a correlation of 0.97. Based on these results, the distinction of light physical activity walking and moderate physical activity can be made adequately.

Important to mention is that Verschuren et al.(7) found that people with stroke their energy expenditures don’t exceed the 1.5 MET in sedentary behavior. Additionally walking at a low pace is below the 3.0 MET and is therefore correctly classified as light physical activity. As shown in the study of Oomen et al. the Activ8 is able to distinguish between walking on a low speed and faster speed. Additionally, our sample had minor stroke with a relatively high walking speed which indicates in general normal physical abilities.

Additionally, in general the Activ8 has specific characteristics that are important in detecting movement behaviors during waking hours and estimate energy expenditures. The thigh is a good location to wear an accelerometer for activity recognition and intensity levels. The activ8 used an activity specific estimation of energy expenditure by means of an intensity factor(8,9). Activity specific estimation methods are found to be better compared with count based estimation methods and activity specific estimation methods using a chart with the metabolic equivalent of task(10).

Based on the studies as mentioned above we are convinced that the Activ8 is able to classify the energy levels correctly.

**Reviewer’s comment:**
Abstract results: I would add if possible which subgroups are identified since this is part of the objectives of the study.

**Authors’ response:**
We agree with the reviewer that this is important information however due to the amount of words it isn’t possible to add this in the abstract.

**Reviewer’s comment:**
Introduction
Your assumptions are contrary to what is reported in the literature, particularly in the literature review of English et a et Rand et al l, which clearly shows that people with stroke tend to be more sedentary. In addition, we observe in the literature and in our clinical experience a strong association between physical activity level and functional abilities, mood disorders, self-efficacy that you do not address at all in your introduction..

**Authors’ response:** We agreed with the reviewer that we didn’t mention factors that are associated with movement behavior outcomes. Therefore we have added following paragraph:

*“Currently, only a few associations are known with regard to movement behavior outcomes. Lower walking speed and walking capacity, balance problems, presence of depression and poorer quality of life associated with accelerometer activity counts*(11)*. Additionally, higher age, being a man, higher cardiorespiratory fitness, lower levels of fatigue, higher level of self-efficacy, presence of depression and higher health related quality of life were factors associate with higher levels of PA*(12)*. Lower walking speed was found to associated with higher amount of sedentary time and long prolonged bouts*(13)*, less functional independence with high amounts of sedentary behavior and prolonged bouts, stroke severity with high amounts of sedentary behavior and age with more prolonged sedentary behavior*(14)*. Although these studies provide preliminary information deeper understanding of factors related with single movement behavior outcomes is needed.”*

**Reviewer’s comment:**
Methodology
Design and participant
How can you justify including only stroke survivors with such a high level of autonomy? This is excessively regressive and unrepresentative of strokes treated in rehabilitation unit.

**Authors’ response:**
We agree with the reviewer that our sample is not representative for the people with stroke in a rehabilitation unit. Due to improvements in acute in the Netherlands about 70% of the populations will be discharged to the home-setting. Within this part of the population 50% of the people won’t get any physiotherapy at all. However, also in people with minor strokes secondary prevention is highly important. In literature it was found that twenty percent of the people with minor stroke were disabled. Disability was found to associated strongly with recurrent stroke, myocardial infarction, and major bleeding(15). In total 16% was found to decline from a level of independence in ADL to a level of dependence in ADL(16). After TIA or minor stroke risk of myocardial infarction and recurrent stroke are respectively 1.67% per year and 4.26% per year(17). For example in the first five years after TIA or minor stroke 12% had a recurrent stroke and 10% died. Recurrent events occur even in those who received excellent evidence-based care (18). Unfortunately, rehabilitation outcomes after a second stroke as well as physical and cognitive outcomes are poor (19). Secondary prevention after first-ever stroke is therefore of paramount interest also in stroke survivors with high levels of autonomy. Especially when considering that the estimated potential of reducing recurrent events is 80% (20). Therefore, we included in this study only people who were discharged immediately to the home-setting since this is >70% of the total population.

We mentioned this in our discussion part: *“Additionally, the participants in this study had relatively minor stroke symptoms but nonetheless were highly sedentary, proportion of the sample was inactive.”*

And

*“Last, our study included only participants who were directly discharged to the home-setting. Since, the majority of this population had minor stroke symptoms the results are not generalizable to a more severe stroke population that received inpatient rehabilitation first. However, our findings emphasize the importance of movement behavior changes since our sample had less severe stroke symptoms but still presented high levels of SB and low levels of MVPA.”*

**Reviewer’s comment:**
Measurements and procedures :
Specify that the evaluation procedures follow this paragraph, otherwise the reader may be frustrated.
Accelerometer
The absence of validation of activ8 in the stroke subject in terms of energy expenditure is a major limitation for the interpretation of the activity intensities reported by the device. Indeed, these intensities are based on the energy expenditure for the activity which is strongly different between healthy individuals and undivided AVC.

**Authors’ response:**
We explained in our previous comment why we think the activ8 is a accurate tool especially in this population with high levels of autonomy. We agree with the reviewer that the energy expenditure would probably deviated in more severe stroke survivors compared to healthy subjects. However, our sample included a population with high levels of autonomy.

**Reviewer’s comment:**
Results :
What was the number of refusals to participate in the study? Wouldn't the patients who agreed to participate in the study be those who consider themselves the least sedentary or inactive? A flow chart seems necessary to inform the reader about these elements.

**Authors’ response:** We have added all information we have about refusal. We added this in the results section.:

*“In total, 180 people with stroke agreed participation when discharged from the hospital to the home-setting. With twenty persons it was not possible to make an appointment within three weeks, fifteen refused further participation, three were unable to contact, one was to ill and one died before our visit.”*

**Reviewer’s comment:**
What was the method used to define the different sedentary classes (low/high)? Is this related to the statistical analysis you describe in the method section? What is the clinical relevance between these 2 separate categories of 2 hours of low-intensity activities when both are sedentary by definition. Same for the activity categories (high, moderate, inactive)...
The authors provide part of the answer in the results paragraph but this is not the place for that. It would be necessary to place this information back in the method section. Moreover, to my knowledge, I do not believe that the clinical relevance of this type of category has been demonstrated in individuals with stroke.

**Authors’ response:** As described in the statistically analysis paragraph in the method section latent class growth analysis was used to identify subgroup trajectories. Using this analysis the most differentiating groups can by identify. To determine the characteristics associated with a single subgroup trajectory, logistic regression analyses were performed. However, if we found over two groups they were merged into two clinically relevant subgroups. The relevance of the amount of sedentary time is high as was found in the recently published study by Ekelund et al(21). This is a large study (harmonized meta-analysis) including over 30.000 people. The study found that time spend in sedentary behavior has a dose response association. The hazard ratio for all-cause mortality increases especially when people spend > 9 ½ hours in sedentary behavior.

**Authors’action:** We removed a sentences within the results section because this was a repetition of the method section.

**Reviewer’s comment:**The last paragraph of the results are, in my opinion, extremely interesting and need to be developed because there is a strong stake in knowing what are the associations between sedentary behaviours, autonomy and social participation scores and mood disorders. The assessment of possible changes in sedentary behaviours and physical activity levels associated with these different dimensions that you have assessed (impairments, activity, participation) as well as those that may or may not benefit from physiotherapy would be all the more original.
To be honest, I expected the authors to establish the subgroups on the clinical characteristics of the participants and not on this type of statistically constructed categories. This limits its clinical interest.

**Authors’ response:** We agree we that the reviewer that it is an interesting research question to establish subgroups based on the outcomes. However, this is another research question. We want to investigate if there were subgroups who had dynamic (e.g. increased MVPA or decreased sedentary behavior) trajectories. Now we know that there are no dynamic subgroups for none of the movement behavior outcomes. Since we did not found change in movement behavior outcomes, movement behavior patterns can be identified by measuring the minimum number of days to acceptable reliability(22).

**Reviewer’s comment:**
Discussion
Is the high value of MVPA in these individuals not related to the particular profile of patients you have included: undivided with very little neuronal sequelae?

**Authors’ response:** Based on the results we cannot confirm the hypothesis. More research will be needed to objectify differences between less and more severe stroke survivors.

**Reviewer’s comment:**
Table 1 :
I don't think we can report the SIS and BBS values on average and SD since they are not continuous variables.

**Authors’ response:**
Initial we presented means and standard deviations for all interval and continues data to improve readability for the reader. However, following the results we should have provided the median and IQR. We changed this in table 1.
